# REAGERA-dementia: study protocol for the validation of screening instruments to detect abuse of people with dementia

**DOI:** 10.1186/s12877-025-06291-z

**Published:** 2025-08-25

**Authors:** Johanna Simmons, Elida Floberg, Christina Casselgren, Björn Westerlind, Jonas Sandberg, Katarina Swahnberg, Katarina Nägga, Mikael Ludvigsson, Linda Johansson

**Affiliations:** 1 Department of Geriatrics and Palliative Medicine, Department of Health, Medicine and Caring Sciences, Linköping University, Linköping, Sweden; 2 Department of Internal Medicine and Geriatrics in Eksjö, Region Jönköping County, Eksjö, Sweden; 3 Department of Health, Medicine and Caring Sciences, Linköping University, Linköping, Sweden; 4 Department of Geriatrics, County Hospital Ryhov, Region Jönköping County, Jönkoping, Sweden; 5 Department of Nursing Science and Institute of Gerontology, School of Health and Welfare, Jönköping University, Jönköping, Sweden; 6 Department of Health and Caring Sciences, Linnaeus University, Kalmar, Sweden; 7 Department of Psychiatry in Linköping, Department of Biomedical and Clinical Sciences, Linköping University, Linköping, Sweden; 8 Institute of Gerontology, School of Health and Welfare, Jönköping University, Jönköping, Sweden

**Keywords:** Elder abuse, Abuse of older people, Dementia, Neurocognitive disorder, Caregiver burden

## Abstract

**Background:**

Abuse of older people is common and people living with dementia are at high risk of being subjected to abuse. This study protocol describes the development and procedure to test the validity of two new screening instruments to detect abuse of people with dementia: 1) The REAGERA-S20, intended to be answered by people with dementia themselves, including questions about their abusive experiences, and 2) The REAGERA – N, intended to be answered by the next of kin of people with dementia, including questions about their own exposure to abuse, their own perpetration as well as the witnessing of abusive acts. In addition, the Risk on Elder Abuse and Mistreatment Instrument (REAMI) is translated into Swedish within the project and will be validated. Finally, qualitative interviews are conducted to explore the experiences of abuse from the perspective of both the person with dementia and the next of kin.

**Method:**

People with mild to moderate dementia (*n* = 80) and their next of kin (*n* = 80) are recruited at health and social care facilities providing care to people with dementia. In cases of severe dementia, only next of kin is included. At the time of data collection, participants fill out the instruments and are thereafter interviewed, first separately and then jointly by researchers about their abusive experiences. The interviews are used as a gold standard for calculating the properties of the instruments, e.g., sensitivity and specificity, but will also be used for qualitative analysis concerning the experiences of abuse. Health and social care professionals that are well acquainted with the participants fill out the REAMI and results will be validated using the results of the interviews as the gold standard.

**Discussion:**

This study protocol describes a research project that aims towards a comprehensive identification and understanding of abuse of people with dementia by including the perspective of both people with dementia themselves, their next of kin and professionals. If the instruments are found to be reliable they can be used to detect abuse in people with dementia. Also, the qualitative interviews with participants will enhance our understanding of abuse of people with dementia.

**Trial registration:**

ClinicalTrials.gov Identifier: NCT06659822. Retrospectively registered on October 25 2024.

**Supplementary Information:**

The online version contains supplementary material available at 10.1186/s12877-025-06291-z.

## Background

Abuse of older people (AOP; also, widely known as elder abuse) is a pervasive human rights and public health problem worldwide [47]. It has been estimated that one in six community dwelling older people globally has been subjected to some form of abuse during the past 12 months [48]. Older people with high functional dependency or cognitive decline are especially susceptible to abuse [5, 15, 25, 40, 49]. Hence, the prevalence of abuse of people with dementia is often found to be considerably higher than for the general population of older people and has been reported at around 50% in international studies, though varying considerably due to methodological differences [16, 45].

There are inherent difficulties in studying abuse of people with dementia who may have reduced cognitive capacity to self-report experiences of abuse. Therefore, in some studies, formal or informal carers are asked to either give proxy reports or to report on their own perpetration of abuse [21, 49]. However, a previous study from China found considerable variations in the reported prevalence of AOP depending on whether the informant was an older adult, their family caregiver or attending medical professionals [16]. In the same study from China, higher prevalence of abuse was reported by care recipients than caregivers [16]. Even so, previous studies found that family carers of people with dementia are willing to report perpetrating abusive acts. For example, Cooper et al. found that 52% of family caregivers reported abusive behaviours towards people with dementia [8]. Likewise, a Norwegian study found that 60% of staff in nursing homes reported perpetration of one or more instances of abuse in the past year and 76% reported witnessing such incidents [2]. That estimate is similar to a global review and meta-analysis reporting that 64% of staff in institutional settings admitted perpetrating AOP in the past year [49].

In this project, we use the World Health Organization definition of AOP and consider it to be *"a single or repeated act, or lack of appropriate action, occurring within any relationship where there is an expectation of trust which causes harm or distress to an older person”* [44]. AOP includes physical, emotional, sexual and financial abuse as well as neglect and has repeatedly been associated with increased ill health as well as higher healthcare consumption and placement in nursing homes [12, 28]. Considering that many older people come in contact with health and social care professionals it has been suggested that such professionals should routinely ask older people questions about abuse [11]. However, a large proportion of professionals are inexperienced in this field and lack education about AOP [32]. Also, though professionals are in a position to detect abuse, it may go unnoticed due to difficulties in identifying abusive acts, which often depend on the interpretation of the current context and situation [26].

Screening instruments concerning abusive experiences may be used as tools to facilitate professionals to ask questions. During the last few years, several screening instruments for AOP have been constructed and validated worldwide, but there is no consensus about a gold standard [17, 42]. Only few instruments have been validated specifically among people with dementia, one being the Modified Conflict Tactic Scale [7]. There are also instruments directed at caregivers, e.g. the Caregiver Abuse Screen [36]. Other instruments are intended to be used by health or social care professionals to estimate the risk of an older person being subjected to abuse, e.g. the Risk on Elder Abuse and Mistreatment Instrument (REAMI) [10] and the Expanded Indicators of Abuse (E-IOA) [6].

Only one screening instrument related to abusive experiences has previously been validated among older people in Sweden, the REAGERA-S (Responding to Elder Abuse in GERiAtric care-Self administrated), which was developed and validated by our research group [39]. The instrument has since been independently tested and evaluated by social workers to screen older people for experiences of abuse or previous life-course experiences of abuse [19]. One limitation to the REAGERA-S is that it is not adapted or tested among people with dementia. Also, there are no available validated Swedish screening instruments concerning abuse of older people that are directed at caregivers or relatives.

The overall aim of the research project described in this study protocol is to validate screening instruments that can be used to detect abuse of people with dementia. In addition, we will explore experiences of abuse from the perspective of both people with dementia and their next of kin. The specific aims are:

1. To assess the validity of two new self-administered screening tools to detect AOP among people with dementia: the REAGERA-S20 (20 for 20 items) directed at people with dementia and the REAGERA-N (N for next of kin) directed at the next of kin.
2. To translate and validate a Swedish version of the REAMI that can be used by professional caregivers to estimate risk of maltreatment and abuse of people with dementia.
3. To explore experiences of abuse among people with dementia and their next of kin.

## Methods and analysis

### Development of instruments

**Fig. 1:**
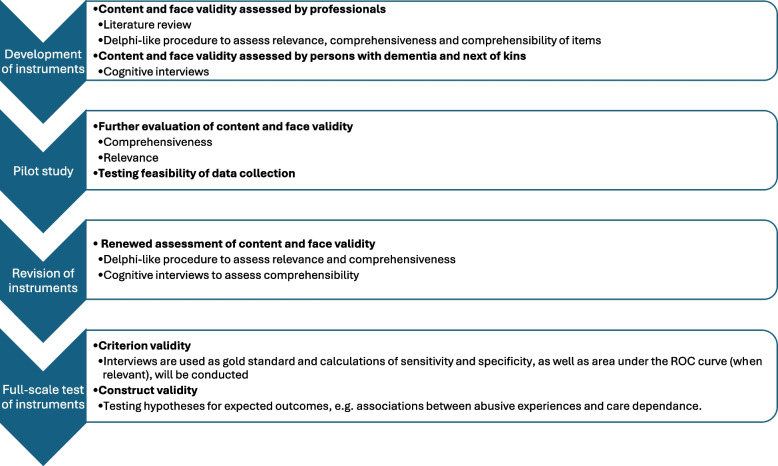
Overview of the methods and procedures used to develop and evaluate the REAGERA-S20 AND REAGERA-N

An overview of the methods and procedures used to develop and evaluate the REAGERA-S20 and REAGERA-N are presented in Fig. 1 and presented in full as follows:

The first versions of the REAGERA-S20 and REAGERA-N were constructed by members of the research team after a review of existing screening tools. The REAGERA-S20 was largely constructed based on the REAGERA-S [39]. However, we also reviewed other instruments to make sure that important aspects of AOP were not missed, e.g., the modified conflict tactics scale [41], ED-EMATS [14] and the Swedish instrument FREDA [33], used in screening for intimate partner violence in younger ages. The first version of REAGERA-S20 contained questions about life-course experiences of psychological, physical, sexual or financial abuse and neglect. The first version of the REAGERA-N constituted two parts: part one contained questions about own exposure to abuse and own perpetration of abuse and part two included questions about witnessing abusive behaviour from third parties. The first part of the REAGERA-N was inspired by the Caregiver Abuse Screen [36] and the second part about witnessing abusive behaviours was largely inspired by the REAGERA-S [39]. The research team, several of whom have extensive experience of working as geriatric consultants in memory clinics, then met to discuss and revise the items in the first versions of the instruments. Efforts were made to keep questions short and make the language precise to assist the intended users—people with mild to moderate dementia and their next of kin.

Thereafter, a procedure similar to the Delphi technique [43] was used to assure face and content validity. In total, 22 professionals and researchers with experience in working with abuse of older people, intimate partner violence and in care services for people with dementia, as well as representatives from a women’s shelter for older people, were asked to rate the items in REAGERA-S20 and REAGERA-N concerning comprehensibility and relevance on a scale ranging from one to seven where seven indicated the highest level of comprehensibility and relevance. They were also asked to assess comprehensiveness of the instruments by suggesting whether more items covering areas of abuse not included in the initial set of questions should be added. The items were thereafter discussed and revised by the research group and the same professionals were asked to again provide their input. In the second round, 14 participants again rated the items for comprehensibility and relevance and the results were satisfactory with most participants rating the items at six or seven.

In the next step, cognitive interviews were performed with two people with dementia and two next of kin to people with dementia. In the cognitive interviews both think aloud technique and probing were used, i.e., participants were asked to answer the questionnaire while reasoning how they interpret the questions and in addition probing questions were asked about how the participant interpreted the items [1]. The procedure was used to assure comprehensibility and face validity of the items by end users. People with dementia had problems reasoning their thinking when answering the questions. Instead, they were asked to fill out the questionnaire and ask the interviewer if they had trouble understanding the items. If found to be hesitant, the interviewer also prompted participants to elaborate on reasons for hesitation. Overall, the items were well understood and the interviews only led to minor revisions of items.

### Pilot study

In 2022, a pilot study testing the instruments as well as feasibility of the intended data collection procedure was carried out. Participants were recruited from two outpatient memory clinics in Sweden and were included in the study in pairs, i.e. a person with dementia and their next of kin. Inclusion criteria for participants were dementia diagnoses of mild to moderate stages. The degree of cognitive decline was assessed with the Global Deterioration Scale (GDS) [37]. The GDS assesses the severity of dementia and defines the different stages of cognitive decline on a scale from one (no cognitive decline) to seven (very severe cognitive decline). Stage four and five are roughly equivalent to mild dementia and moderate dementia, respectively. The GDS considers both the level of functional and cognitive decline [37]. Next of kin was a relative or close friend, well acquainted with the person with dementia. Most often, the next of kin was a partner but there was no specific requisite concerning the relationship. Exclusion criteria were dementia of severe degree, severe psychiatric illness or significant communication difficulties due to e.g. aphasia or not speaking Swedish which made it impossible to be interviewed. Eligibility to participate in the study was assessed by healthcare professionals working at memory clinics in conjunction with the researchers.

In total, ten people with GDS score four and 14 people with GDS score five were included in the pilot study together with a next of kin. Participants attended the memory clinic for one research visit during which they separately filled out the instruments and thereafter were interviewed, first separately by two different researchers and then jointly, about possible abusive experiences. The interviews were recorded, transcribed verbatim and used as a gold standard to which the instruments were eventually assessed.

The content of all interviews was discussed at meetings with the research group and a consensus was reached for each participant as to whether they should be classified as exposed or non-exposed to AOP as well as exposed or non-exposed to abuse earlier in life. The WHO definition of AOP [44] was used as a reference for discussions and, if needed, the Centers for Disease Control and Prevention’s (CDC) more elaborate definition and core element concerning AOP [23] was also consulted. Most cases that were misclassified by the instruments were cases classified as negative by the instruments but classified as positive in the interview either for psychological AOP or abuse from earlier in life. Hence, there was a need to improve the sensitivity of the instruments concerning these aspects.

### Revision of instruments

As a result of the pilot test, a continuing discussion with health and social care professionals and the simultaneous publication of an independent evaluation [19] of the original version of REAGERA-S [39] in social care, a substantial revision of the instruments was performed. In particular, the structure of the instruments was changed in two ways. 1) The life-course perspective originally used for all items was replaced by one part about abusive experiences during the past 12 months and another part about abusive experiences earlier in life. 2) A few introductory screening items to assess risk of abuse were included to limit the number of questions used for those with low risk of abuse.

Also, the first versions of the instruments were only targeted at people with dementia and their relatives, which was mentioned in the introductory text. To simplify for end users, i.e. making it possible for health and social care workers to use the same instrument regardless of cognitive status of the respondent, the introductory text was changed to fit next of kin to all adults with some form of care dependence (REAGERA-N) and all adults (REAGERA-S20) respectively.

The revised versions of the instruments were thereafter evaluated in a renewed Delphi-like procedure. In total, 33 professionals and researchers assessed the instruments’ comprehensiveness and rated the items on relevance and comprehensibility using an online questionnaire. The items were generally given high scores and only minor changes to the instruments were made because of the responses.

As a final step, the two instruments were again tested with cognitive interviews [1], using the same procedure as previously described. This time, six people with dementia and six next of kin were included. Again, minor changes to the wordings were made in accordance with the results of the interviews.

### Final version of the instruments and intended use

The final versions of REAGERA-S20 and REAGERA-N can be found in the supplement.

REAGERA-S20 contains three different parts. Part 1) Three introductory screening questions to assess risk situations for abuse. Part 2) Twelve questions about exposure to different forms of abuse during the past 12 months. Part 3) Five questions about previous life-course experiences of abuse.

The REAGERA-N consists of four different parts. Part 1) Four introductory screening questions to assess risk situations for abuse. Part 2) Four questions about own exposure to abuse by the person with dementia and six questions about own perpetration of abuse during the past 12 months. Part 3) Seven questions about witnessing abusive experiences towards the person with dementia from third parties, e.g. other relatives or care professionals, during the past 12 months. Part 4) Five questions about experiences of abuse from earlier in life for the person with dementia.

For both instruments, the intended use is that positive responses to the introductory screening questions in part one should lead to using the second part of the questionnaire. Part three in the REAGERA-S20 and part four in the REAGERA-N are intended to be used when a life-course perspective is deemed relevant. The questions in part three in REAGERA-N, about witnessing abusive experiences, may be used when an older person is not able to answer questions themselves, e.g. due to severe cognitive impairment or poor physical functioning.

### The REAMI

The REAMI is a 22 item questionnaire originally developed to be used by professionals to examine risk of AOP [10]. The questions cover risk factors of the older person and environment as well as signals of abuse and mistreatment. Possible answers to all items are “I completely disagree,” “I rather disagree,” “I rather agree” and “I completely agree”. The total score is calculated and converted to a risk estimation of the older person being subjected to abuse (“no risk,” “low risk,” “moderate risk” and “high risk”).

The English version of the instrument was translated into Swedish by three of the participating researchers and then translated back into English by two professional translators. A meeting between the researchers and translators was thereafter held to reach consensus about the best possible Swedish translation. The Swedish translation was then tested using cognitive interviews with six professionals working with providing care to people with dementia. Minor adjustments of the wording of questions were made after the interviews and an introductory text was added to enhance understanding of the instrument. All changes were discussed with the researchers who originally developed the instrument [10].

### Other measurements

To further characterise the participants and explore factors that could potentially be associated with abusive experiences, the following questionnaires are also included in the data collection.

- UCLA 3-item loneliness scale, measuring subjective feelings of loneliness as well as feelings of social isolation [38], is used to capture feelings of loneliness among next of kin. It has previously been translated and used in a Swedish context [27] and the 3-item version has been found to be a reliable and validated measure of loneliness [24].
- Zarit Burden Interview (ZBI) measures the perceived impact on the caregiver of providing care [50] and has previously been validated in Swedish [22]. The brief 12-item version used in the current study has been tested with satisfactory results [20].
- Functional activities questionnaire (FAQ) measures instrumental activities of daily living (IADLs). The FAQ is administered to a lay informant, in this study the next of kin. It consists of ten items regarding the ability of the person with dementia to perform daily tasks needed when living independently [35] and is used in clinical practice in some memory clinics in Sweden.
- The Neuropsychiatric Inventory Questionnaire (NPI-Q) measures the frequency and severity of neuropsychiatric symptoms for the patient with dementia [9]. The Swedish version of the questionnaire is answered by next of kin in the current study.
- The Tilburg Frailty Indicator (TFI) consists of items concerning physical, psychological as well as social aspects of frailty and in this study is used to capture the frailty status of the people with dementia. It has been validated with satisfactory results both internationally and in Sweden [18, 29].
- Carer’s Assessment of Managing Index (CAMI) is a 15-item questionnaire used for assessing caregiver coping strategies and has previously been used in a Swedish study [13, 34].
- Relationship rewards is a measure of relationship satisfaction before and after onset of illness [46]. In the current study, the questions are answered by both the person with dementia and next of kin.

### Full scale test of instruments

#### Participants and procedure

Participant recruitment started in spring 2023 and is planned to continue until the end of 2024. Participants are recruited from three outpatient memory clinics in Sweden, from primary healthcare centres and municipalities caring for people with dementia.

Inclusion criteria for people with dementia are a dementia diagnosis and having a cognitive decline assessed as GDS 4 or 5. Inclusion criteria for next of kin is being next of kin to a person diagnosed with dementia, with the degree of cognitive decline assessed as GDS 4–7. Exclusion criteria for both people with dementia and next of kin are severe psychiatric disorder, not being able to answer the questionnaires or participate in an interview, e.g. due to insufficient proficiency in Swedish, aphasia, hearing loss or similar. In addition, people with dementia experiencing symptoms that may be exacerbated by study participation or make results less reliable (e.g. paranoia) are excluded.

Inclusion and exclusion criteria are assessed by professionals working at each centre recruiting participants and, when needed, in conjunction with a member of the research team. Written information about the study as well as about available societal support for victims of AOP is handed out in conjunction with contact with a care provider at the clinic or municipality. A researcher thereafter contacts potential participants and if needed gives additional information about the study. Subsequently, a time of interview is arranged for those agreeing to participate. Interviews are mainly conducted at the healthcare facilities where participants are recruited but if needed home visits can also be provided. A prerequisite for a house call is that interviews can be carried out in two separate rooms.

**Fig. 2:**
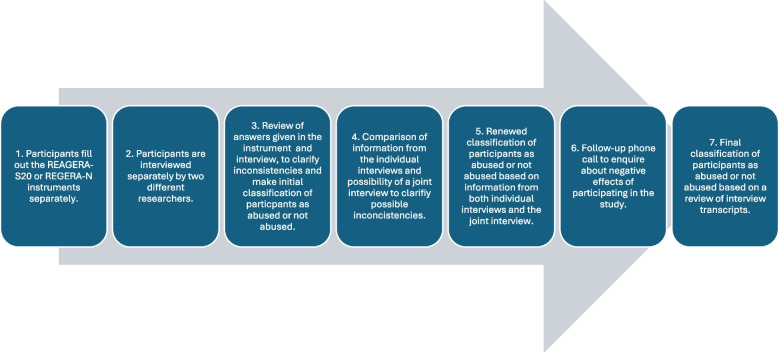
Overview of the data collection procedure

An overview of the data collection procedure is given in Fig. 2 and presented in full as follows:

1. At the research visit, participants are placed in different rooms and individually answer the questionnaires REAGERA-S20 and REAGERA-N respectively. The next of kin are also asked to complete the following scales and questionnaires: the UCLA loneliness scale [24], the ZBI [20], the FAQ [35], the NPI-Q [9], the relationship reward questionnaire [46] and the CAMI [13, 34].
2. Semi-structured interviews are then conducted with the person with dementia and their next of kin separately and simultaneously. The two researchers conducting the interviews are blinded to the answers given by the participants in the questionnaires. The interview starts with general background questions and moves on to questions about exposure to abuse. Initially, open-ended questions about negative experiences and difficulties in the relationship are asked. Thereafter, a more structured part of the interview is conducted in which specific questions about physical, psychological, sexual and financial abuse as well as neglect are asked. A digital interview guide is used, in which the researcher records the answers given by the participant to questions about different forms of abuse. If the participant reveals any kind of abusive experiences, an in-depth qualitative interview about the experiences is conducted at any time during the interview. Interviews are recorded and transcribed verbatim which allow for a later review of content as well as qualitative analyses. For the person with dementia, the questions in the Tilburg Frailty Indicator [18, 29] and relationship reward questionnaire [46] are also included in the interview.
3. At the end of the individual interviews, the researchers check the answers given by the participant in REAGERA-S20 or REAGERA-N and compare them to the responses recorded in the digital interview guide during the interview. This allows for clarification of inconsistent responses and a preliminary classification of the person with dementia as abused or not abused based on the information provided in each individual interview.
4. After the individual interviews have been completed, the two researchers compare the results from each interview. If needed, and with permission obtained individually from the participants, a joint concluding interview is conducted. This interview again allows for clarifying possible inconsistencies.
5. After the interviews are completed, the two researchers together make a new classification of abuse status, considering information from both interviews. It is also noted if abuse earlier in life happened before the age of 18, between 18 and 60 or after the age of 60. In addition, the type of relationship with the perpetrator is noted. This information is collected to be able to understand false negatives, i.e. what types of abusive experiences are not captured by the REAGERA-S20 and REAGERA-N.
6. One follow-up phone call is made after the interview to inquire about negative effects of participating in the study or if participation has given rise to any thoughts or questions. If there are indications of emotionally stressful or traumatic reactions of participating in the interview, relevant referrals to societal resources can be made at this stage.
7. All interviews are recorded and transcribed verbatim. Thereafter, a review of the classification of participants as abused or not abused made during the interviews is conducted, using the transcript as a reference. The review is made by at least two researchers to make sure that similar classifications are made for all participants concerning kinds of experiences that constituting abuse. The final classification will be used as the gold standard against which the validity of the screening instruments is measured. If there is doubt whether an experience should be classified as abusive or not, discussions will be held within the research group and the Toronto Declaration [44] and the CDC definition and core elements of elder abuse [23] will additionally be used for the assessment. The following classification is used:
  For the person with dementia
    Reporting or not reporting abusive experiences during the past 12 months.
    Reporting or not reporting abusive experience earlier in life, i.e. more than 12 months ago.
  For the next of kin
    Reporting or not reporting own exposure to abuse by the person with dementia during the past 12 months.
    Reporting or not reporting own perpetration of abuse towards the person with dementia during the past 12 months.
    Reporting or not reporting that the person with dementia has been exposed to abuse by another person during the past 12 months.
    Reporting or not reporting that the person with dementia has been exposed to abuse earlier in life, i.e. more than 12 months ago.

When a next of kin is included alone, steps 1–3 and 6–7 are conducted.

The need for societal support for both the person with dementia and the next of kin will be evaluated at the end of each individual interview as well as at the joint interview and follow up call. This gives repeated opportunities to provide relevant referrals if needed, both in relation to potential AOP and in relation to potential stressful or traumatic reactions in relation to participating in the study.

After the interviews have been completed, the REAMI is sent electronically to a health or social care provider who has knowledge about the person with dementia, and they are asked to fill it out.

### Sample size

Ten of the 24 people with dementia included in the pilot test (42%) were classified as having experienced some form of abuse during the past 12 months. This number is similar to previous research concerning abuse of people with dementia [15, 45] and was used for the sample size calculation. The sample size was calculated from tables presented in a study by Bujang and Adnan [4]. In their study, they constructed a formula for estimating sample size for different combinations of estimated prevalence, sensitivity and specificity for screening and diagnostic instruments. For screening instruments, the sensitivity of the instrument is more important than the specificity. According to Bujang, 67 participants are needed to test a screening instrument that has 80% sensitivity at 40% prevalence rate. To assure that the sample size is sufficient, if the sensitivity or prevalence rate is smaller than expected, we aimed at including 80 pairs of participants, i.e. 80 people with dementia and 80 next of kin.

### Statistical analysis plan

#### Aim 1: Testing the validity of REAGERA-S20 and REAGERA-N

Primary outcomes:

1. Criterion validity will be assessed by calculating sensitivity, specificity, positive and negative likelihood ratio as well as positive and negative predictive value of each tool using the final classification made after interviewing both the person with dementia and the next of kin, i.e., the previously described step 7. Only participants for which data is available from both the person with dementia and next of kin will be included in the analyses. For REAGERA-S20, one or more positive answers in part two will be classified as reporting abusive experiences during the past 12 months. One or more positive answers in part three will be classified as abusive experiences earlier in life. For REAGERA-N, one or more positive answers to items concerning perpetration of abuse by next of kin in part two (no 1,3,6–8, 10) or any of the items in part three about witnessing abusive acts will be classified as abuse of the person with dementia during the past 12 months. Affirmative answers to items in part four will be classified as abusive experiences from earlier in life.
2. Test the predictive value of the introductory screening questions in part one of each instrument to identify abusive experiences as identified in part two as well as the final classification of participants as abused or not abused. The introductory screening questions are answered on a three-point ordinal scale (“never,” “sometimes,” “often”). A sum score will be calculated for all items and the best threshold for discriminating between abused and not abused participants will be calculated using a Receiver Operating Curve (ROC) Analysis. We will also examine if one item or a combination of specific items are most likely to predict abusive experiences.

Secondary outcomes:

1. Sensitivity, specificity, positive and negative likelihood ratio as well as positive and negative predictive value for REAGERA-S20 using only information given in the individual interview with the person with dementia as the gold standard.
2. Sensitivity, specificity, positive and negative likelihood ratio as well as positive and negative predictive value of REAGERA-N for all participants, e.g. also including next of kin to people with GDS score ≥ 6 or people with dementia that were excluded for some other reason. The interview with next of kin will be used as a gold standard and the following classification of abuse according to REAGERA-N will be made: a) one or more positive answers to items 2,4,5 and 9 in part two about abusive behaviours by the person with dementia will be classified as next of kin’s own exposure to abuse during the past 12 months, b) one or more positive answers to items 1,3,6–8, 10 about abusive behaviours towards the person with dementia will be classified as own perpetration of abuse during the past 12 months, c) one or more positive answers to the items in part 3 will be classified as witnessing abusive experiences during the past 12 months and d) one or positive answers in part 4 will be classified as reporting that the person with dementia has been exposed to abuse earlier in life.
3. When there is no established gold standard for a concept, one way to ensure construct validity of a new measurement is to test hypotheses about expected associations with established outcome measurements of related concepts [31]. Care dependence is a well-established risk factor for AOP [5, 15, 25, 40, 49] and we therefore expect to find an association between reporting experiences of abuse and reporting higher scores on ZBI as well as FAQ.

#### Aim 2: Validity testing of the Swedish version of the REAMI

The risk of abuse is assessed on a four-point scale on the REAMI (“no risk,” “low risk,” “moderate risk” and “high risk”). The final classification of participants as abused or not abused will be used as a gold standard and a ROC analysis will be used to investigate the threshold for which the REAMI best can discriminate between abused and non-abused participants. Sensitivity, specificity, positive and negative likelihood ratio as well as positive and negative predictive value will then be calculated for that value. As the REAMI was also included in the pilot study and has not been revised since, this analysis will also include the twenty-four pairs of participants included in the pilot study.

#### Aim 3: Explore experiences of abuse

Individual interviews with both the person with dementia and next of kin, as well as the joint interview, will be used to explore experiences of abuse from different perspectives. Interviews will be transcribed verbatim and analysed using qualitative methods, such as thematic analysis [3].

## Discussion

AOP is a diverse, subjective and contextual experience. Therefore, one major challenge when developing a screening instrument for AOP is to determine how to assess criterion validity since there is no generally agreed upon objective measure of AOP that can be used as the gold standard. Assessment by professional health or social care workers has been used in some previous studies. This is a valid approach but will inevitably miss some cases as there may be no visible signs of abuse, especially considering that the most prevalent form of AOP is psychological abuse [48]. In this study, we use the experience of people with dementia and their next of kin as the gold standard. This also has disadvantages as it relies on the participants’ ability to remember and willingness to share abusive experiences with the researcher in the interview. However, the personal experience of the involved parties is considered as a core element of abuse and, hence, relying on participants’ own testimony was regarded as the best available option for a gold standard.

A second major challenge is to find ways to assess the psychometric properties of a screening instrument for abuse. A recent systematic review of the psychometric properties of instruments used for measuring the prevalence of abuse of older people in community and institutional settings found that internal consistency was the most frequently assessed psychometric property among included instruments, most often assessed by reporting Cronbach’s alpha [30]. However, when constructing a screening instrument for AOP, the task is to construct an instrument that will capture as diverse abusive experiences as possible while using as few questions as possible. Hence, unidimensionality is not sought after and will not be assessed for REAGERA-S20 and REAGERA-N. Rather, the focus of this project is to assure face, content and criterion validity of the instruments.

There is no classification of severity of abuse in the REAGERA-S20 or REAGERA-N. Rather, when used in for example health or social care, all affirmative responses should be followed up with a discussion about what has happened and what can be done to address the situation.

Due to both ethical and methodological difficulties, people with dementia are often excluded from giving first-hand information in research concerning AOP. However, when doing so other ethical dilemmas and methodological difficulties are introduced in studies. Excluding people with dementia from studies on abuse may lead to pivotal misunderstandings and gaps in knowledge concerning abuse towards one of the most vulnerable groups of older people.

Though we consider including people living with dementia as a major strength of this project it also introduces challenges. The most important of which are ethical considerations concerning participants’ safety and difficulties assuring informed consent. Several measures have been taken to limit these potential risks throughout the data collection. People with severe cognitive decline (GDS 6 or more) are excluded from the study since it is difficult to assure that this group understands the research objective and can give informed consent. Inclusion and exclusion criteria are assessed by health and social care workers who distribute information to potential participants. If a person is known to experience paranoia, anxiety or other symptoms that may be exacerbated by study participation or make the results less reliable, they are excluded from study participation. Further, all interviews are conducted by medical professionals experienced in working with people with dementia, i.e. geriatric consultants, a final-year resident in geriatric medicine and a nurse. Informed consent is considered a continuum during data collection. If the researchers find that participants have difficulties understanding the context and the previously given informed consent is perceived as not really informed, the data collection is aborted and the participant is excluded from analysis. Also, all participants have the choice not to answer specific questions in the instruments, refuse to answer questions in the interview or abort the data collection themselves.

Next of kin may be included alone but people with dementia are only included together with their next of kin to assure that someone is there after data collection and, if needed, can assist in seeking help. This decision was made to assure safety for participants but may result in a selection bias. It is likely that older people suffering with severe forms of violence from a next of kin may choose not to participate. However, previous research [8] as well as the results of our pilot study suggest that next of kin are willing to disclose perpetrating acts of abuse. Also, in addition to providing a more comprehensive picture of potential abuse by the next of kin, interviewing both the person with dementia and the next of kin may facilitate disclosing other forms of abuse, e.g. by health or social care staff.

People with dementia and their next of kin are first interviewed separately and then together. To further assure safety and confidentiality for participants, the individual interviews are concluded with a discussion about what parts of the interviews may be shared in the following joint interviews. An assessment of the need to refer participants to relevant societal support organisations is also made together with participants at the end of both the individual and joint interviews. Participants are also telephoned within a few weeks of the data collection and asked about negative reactions to participating in the study. This provides a second opportunity to make relevant referrals for those reporting experiences of abuse or reporting stressful reactions to participating in the interview. Follow-up phone calls were also made in the pilot study and no severe negative reactions to participating in the study was reported. As an additional safety measure, information about societal support available in relation to AOP is handed out together with the information about the study. This assures that all participants as well as those choosing not to participate has information on where to turn for help if needed.

Data collection is carried out on one occasion. This includes both answering the REAGERA-S20 or REAGERA-N, individual interviews and the joint interview. This choice was made to assure the safety of participants. If the questionnaires were sent home to be filled out beforehand, privacy of answers could not be assured. However, this procedure means that there is very little time between filling out the instruments and participating in the interview. It is therefore likely that participants recall their answers in the questionnaires when the interview is conducted. This may be considered as a limitation because responses in the questionnaire and in the interview are not completely independent. However, the aim of the interview is to reveal all experiences of abuse and, hence, it is rather considered a strength if some participants are prompted to remember and disclose experiences that may otherwise have not come up in the interviews.

## Conclusion

This study protocol describes one of few research projects that aims at a comprehensive identification and understanding of abuse of people with dementia by simultaneously considering the perspective of people with dementia themselves, their next of kin and professionals. It includes testing instruments that can be used to detect abuse in all three populations (people with dementia, next of kin and professionals) as well as qualitative interviews that will enhance our understanding of abuse of people with dementia. All this is essential for future development and societal implementation of interventions for abuse of people with dementia.

## Supplementary Information

Supplementary Material 1.

Supplementary Material 2.

## Data Availability

No datasets were generated or analysed during the current study.

## Abbreviations

AOP: Abuse of older people
REAGERA: Responding to Elder Abuse in GERiAtric care.
ZBI: Zarit Burden Interview
FAQ: Functional Activities Questionnaire
CAMI: Carer’s Assessment of Managing Index
TFI: Tilburg Frailty Indicator
NPI-Q: Neuropsychiatric Inventory Questionnaire
