# Supplementary material for: REAGERA-dementia: study protocol for the validation of screening instruments to detect abuse of people with dementia
Source: BMC Geriatr. 2025 Aug 25;25:660. doi: 10.1186/s12877-025-06291-z (PMC12376475; doi:10.1186/s12877-025-06291-z)
Supplement: Supplementary file 1 — Supplementary Material 1. [file 12877_2025_6291_MOESM1_ESM.pdf]

Supplement 1. REAGERA-S20

Please note that the original language of the REAGERA-S20 is Swedish. The Swedish version has been translated by a professional translator into English and then back-translated into Swedish by a second professional translator. Discrepancies between the original version and the back-translated version was thereafter reviewed by the researchers and when needed discussed with the translators.

Experiences of negative actions

It is quite common to be subjected to negative actions by people close to you, such as relatives, acquaintances or health and social care workers.

Needing help to manage your daily life can lead to stressful situations and put you at greater risk of being subjected to negative behaviour, even by people who care about you.

The questions below are about different negative actions and are asked so that those who need help and support can get it.

1.	Does anyone around you treat you disrespectfully?	**Never**	**Some times**	**Often**
2.	Do you feel uncomfortable with anyone around you?	**Never**	**Some times**	**Often**
3.	Does anyone around you use a harsh tone of voice or treat you rudely?	**Never**	**Some times**	**Often**

Have any of the following happened to you in the past year?		
1. Has anyone repeatedly talked down to you or insulted you?	NO	YES
2. Has anyone repeatedly yelled at or scolded you?	NO	YES
3. Has anyone tried to control what you do or decide who you can see?	NO	YES
4. Have you felt afraid of anyone around you?	NO	YES
5. Has anyone threatened to harm a person you care about or your pet?	NO	YES
6. Has anyone pushed or pinched you?	NO	YES
7. Has anyone hit or kicked you?	NO	YES
8. Has anyone made unwanted sexual comments?	NO	YES
9. Has anyone touched your body without your consent or forced you to perform sexual acts?	NO	YES
10. Have you ever been denied help when you needed it, for example to eat, get dressed or take medicine?	NO	YES
11. Has anyone been heavy-handed or treated you poorly when they were supposed to help you, for example with food, clothing or medicine?	NO	YES
12. Has anyone swindled you out of money or stolen from you?	NO	YES

Negative events earlier in life

Violations, violence and abuse can occur at any time of life.

A person may have been subjected by a relative, such as a parent, a partner or an adult child, or it may have been done by a health or social care worker or another person.

Even if it happened a long time ago, it could affect how a person feels now and reacts in different situations.

The following questions are about things that happened earlier in life. They may have happened when you were a child or an adult.

1.	Has anyone threatened you, humiliated you, tried to tell you what you can do, or subjected you to any other psychological violence?	**NO**	**YES**
2.	Have you been hit, kicked or subjected to other physical violence that you found frightening?	**NO**	**YES**
3.	Has anyone touched your body without your consent, forced you to perform sexual acts or subjected you to any other sexual abuse?	**NO**	**YES**
4.	Has anyone swindled you out of money, exploited you financially or taken control of your finances against your will?	**NO**	**YES**
5.	Have you ever been deprived of the help or assistive devices you needed to manage your daily life?	**NO**	**YES**
