# Supplementary material for: REAGERA-dementia: study protocol for the validation of screening instruments to detect abuse of people with dementia
Source: BMC Geriatr. 2025 Aug 25;25:660. doi: 10.1186/s12877-025-06291-z (PMC12376475; doi:10.1186/s12877-025-06291-z)
Supplement: Supplementary file 2 — Supplementary Material 2. [file 12877_2025_6291_MOESM2_ESM.pdf]

Supplement 2. REAGERA-N.

Please note that the original language of the REAGERA-N is Swedish. The Swedish version has been translated by a professional translator into English and then back-translated into Swedish by a second professional translator. Discrepancies between the original version and the back-translated version was thereafter reviewed by the researchers and when needed discussed with the translators.

One of the most difficult words to translate was the Swedish word “Närstående” used in the original version of the instrument. It has been translated into “next of kin” but it should be noted that the Swedish word entails “next of kin”, “relatives” and other close relationships. It is not restricted to blood relatives. Hence, even though the term “next of kin” is used, the instrument can be answered by anyone with a close relationship to the person with dementia.

The relationship between you and your next of kin

When someone needs help in their daily life, it can affect their relationship with relatives and other people around them and lead to situations that are difficult to manage.

It can lead to people subjecting each other to different types of negative behaviours, even if they do not intend to harm the other person.

The questions below are intended to draw attention to such situations so that those who need help and support to manage their situation can receive it

1. Do you feel limited in your daily life because you need to take care of your next of kin?	**Never**	**Some times**	**Often**
2. Do you find it difficult to cope with your next of kin's mood or behaviour?	**Never**	**Some times**	**Often**
3. Does your next of kin snap at you or treat you poorly?	**Never**	**Some times**	**Often**
4. Do you treat your next of kin in a way that makes you feel guilty later?	**Never**	**Some times**	**Often**

The relationship between you and your next of kin, Part 2			
1. Do you talk down to your next of kin?	Never	Some times	Often
2. Does your next of kin yell at or scold you?	Never	Some times	Often
3. Do you yell at or scold your next of kin?	Never	Some times	Often
4. Do you ever feel afraid of your next of kin?	Never	Some times	Often
5. Is your next of kin ever physically violent, for example by pushing, hitting or kicking you?	Never	Some times	Often
6. Do you ever restrain or otherwise roughly handle your next of kin?	Never	Some times	Often
7. Do you ever refuse to help your next of kin when they need it, for example with food, clothing or medicine?	Never	Some times	Often
8. Do you ever use your next of kin's money, against their wishes, to buy things for yourself or for someone else?	Never	Some times	Often
9. Does your next of kin ever touch your body without your consent or make unwanted sexual comments?	Never	Some times	Often
10. **If your next of kin is your partner:** Are you ever unsure whether your next of kin has consented to sexual acts that you have done?	Never	Some times	Often

Your next of kin's victimisation in the past year

People who need help and support in their daily lives are at greater risk of being subjected to violations, violence or abuse in various situations. They may be subjected to such actions by relatives, health and social care workers or other persons.

Below are some questions about whether you are aware of, or have witnessed, such acts against your next of kin in the past year.

1. Has your next of kin seemed uncomfortable with or expressed fear of someone close to them?	**No**	**Yes**
2. Has anyone repeatedly insulted, belittled or said anything that hurt your next of kin?	**No**	**Yes**
3. Has someone swindled your next of kin out of money or stolen from them?	**No**	**Yes**
4. Has your next of kin been subjected to physical violence, such as being pushed, pinched, restrained, punched or kicked?	**No**	**Yes**
5. Has your next of kin been subjected to any sexual abuse, such as unwanted sexual comments or someone touching your next of kin's body without their consent?	**No**	**Yes**
6. Has your next of kin been denied help when they needed it, for example with eating, getting dressed or taking medicine?	**No**	**Yes**
7. When helping your next of kin, has anyone treated them poorly, for example by being disrespectful or heavy-handed?	**No**	**Yes**

Your next of kin's victimisation earlier in life

Violations, violence and abuse can occur at any time of life. Even if it happened a long time ago, it could affect how a person feels now and reacts in different situations.

A person may have been subjected by a parent, a partner or an adult child, or it may have been done by a health or social care worker or another person.

Do you know whether your next of kin has been subjected to any of the following in the past, whether as a child or as an adult?

1. Psychological violence, such as being humiliated, belittled, threatened or having someone try to tell them what they could or could not do?	**No**	**Yes**
2. Physical violence that your next of kin found frightening, such as being punched or kicked?	**No**	**Yes**
3. Sexual abuse, such as someone touching your next of kin's body without their consent or forcing them to perform sexual acts?	**No**	**Yes**
4. Financial abuse, such as your next of kin being financially exploited or swindled out of money?	**No**	**Yes**
5. Neglect, such as not providing your next of kin with the help or assistive devices they needed to manage their daily life?	**No**	**Yes**
